# Draft Genome Sequences of *Mycobacterium bovis* BZ 31150 and *Mycobacterium bovis* B2 7505, Pathogenic Bacteria Isolated from Archived Captive Animal Bronchial Washes and Human Sputum Samples in Uganda

**DOI:** 10.1128/genomeA.01102-15

**Published:** 2015-10-08

**Authors:** Sylvia I. Wanzala, Jesca Nakavuma, Dominic A. Travis, Praiscillia Kia, Sam Ogwang, Srinand Sreevatsan

**Affiliations:** a College of Veterinary Medicine, Animal Resources and Biosecurity, Makerere University, Kampala, Uganda; b Veterinary Population Medicine Department, College of Veterinary Medicine, University of Minnesota, St. Paul, Minnesota, USA; c Joint Clinical Research Center (JCRC) Mengo, Kampala, Uganda

## Abstract

Bovine tuberculosis (BTB), a zoonotic infection of cattle caused by *Mycobacterium bovis*, results in losses of $3 billion to the global agricultural industry and represents the fourth most important livestock disease worldwide. *M. bovis* as a source of human infection is likely underreported due to the culture medium conditions used to isolate the organism from sputum or other sample sources. We report here the draft genome sequences of *M. bovis* BZ 31150, isolated from a bronchial washing from a captive chimpanzee, and *M. bovis* B2 7505, isolated from a human sputum sample in Uganda.

## GENOME ANNOUNCEMENT

Zoonotic transmission of bovine tuberculosis is a major concern in pastoral settings of the developing world, where animal-human interface is close and HIV prevalence is high. The continued transmission of *Mycobacterium bovis*, which is resistant to pyrazinamide, leads to treatment failures necessitating improved diagnostics to inform national and international tuberculosis (TB) surveillance efforts.

*M. bovis* causes significant economic losses in domestic food-producing animals (1, 2). The extent of human tuberculosis caused by *M. bovis* is largely unknown due to a lack of accurate laboratory testing, especially in resource-limited settings of the world. Human cases of bovine tuberculosis are underreported in these areas, primarily because glycerol, which is used as a carbon source to grow *Mycobacterium tuberculosis*, is not optimal for *M. bovis* growth. This means that *M. bovis* detection in human cultures is markedly reduced with standard media.

*M. bovis* strain BZ 31150 was isolated from an archived bronchial wash sample from a chimpanzee, and strain B2 7505 was isolated from an archived human sputum sample at the Joint Clinical Research Center (JCRC) in Kampala, Uganda. Institutional review board (IRB) approval was obtained from Makerere University, School of Biomedical Sciences, Research and Ethics Committee. Scientific and ethical clearance was also obtained from the Uganda National Council for Science and Technology (UNCST) under reference no. HS 1478.

DNA was subjected to whole-genome shotgun sequencing from a paired-end library, performed using the Illumina HiSeq 2000 platform from the Broad Institute (GA Pipeline version RTA1.17.21.3), with 1.1 M spots yielding 214.9 Mb for *M. bovis* BZ 31150, and 1.8 M spots yielding 356.7 Mb for *M. bovis* B2 7505.

*M. bovis* BZ 31150 annotations performed at the Broad Institute revealed 4,007 proteins, a G+C content of 65.5%, 3 rRNAs, 45 tRNAs, 57 pseudogenes, and 4,118 genes. The assembly method used AllPaths version R48559. Illumina reads were assembled into 141 contigs and 136 scaffolds, resulting in a genome size of ~4.272 Mb, with a G+C content of 65.54% at 24× coverage (http://www.ncbi.nlm.nih.gov/assembly/GCF_000649655.1#/st). Annotation with the PATRIC database (3) resulted in 4,284 coding sequences (CDSs), 3 rRNAs, and 45 tRNAs. There are also 105 pseudogenes. Of the CDSs, 1,129 (26.35%) were annotated as hypothetical proteins, and the remaining 3,155 (73.64%) had putative functions.

*M. bovis* B2 7505 annotations performed at the Broad Institute revealed 3,656 proteins, a G+C content of 65.5%, 3 rRNAs, 45 tRNAs, 1 other RNA, 374 pseudogenes, and 4,079 genes (http://www.ncbi.nlm.nih.gov/genome/161?genome_assembly_id=159783). The assembly method used AllPaths version R48559. Illumina reads were assembled into 243 contigs and 206 scaffolds, resulting in a genome size of ~4.17 Mb, with a G+C content of 65.5% at 31.0× coverage (http://www.ncbi.nlm.nih.gov/assembly/GCF_000649675.1/#/st). Annotation with the PATRIC database (3) resulted in 4,218 CDSs, 3 rRNAs, and 45 tRNAs. There are also 125 pseudogenes. Of the CDSs, 1,126 (26.7%) were annotated as hypothetical proteins, and the other 3,092 (73.3%) had putative functions.

### Nucleotide sequence accession numbers.

The whole-genome sequence of *M. bovis* strain BZ 31150 has been deposited at GenBank under accession no. JKAM00000000. For *M. bovis* strain B2 7505, the whole-genome sequence has been deposited at GenBank under accession no. JKAL00000000.
